# Harnessing the potential of large language models in medicine: opportunities, challenges, and ethical considerations

**DOI:** 10.1097/JS9.0000000000001613

**Published:** 2024-05-15

**Authors:** Zhaohui Zhou, Wenyi Gan, Jiarui Xie, Zeji Guo, Zhiling Zhang

**Affiliations:** a Department of Urology, Sun Yat-Sen University Cancer Center; b The Second School of Clinical Medicine, Southern Medical University, Guangzhou; c Department of Joint Surgery and Sports Medicine, Zhuhai People’s Hospital (Zhuhai Hospital Affiliated with Jinan University), Zhuhai, Guangdong, People’s Republic of China

*Dear Editor,*

As a typical representative of large language models (LLMs), ChatGPT has seen a significant expansion in its medical applications since the release of the GPT-3.5 version in 2022. The model has demonstrated extensive potential in various aspects, including diagnostic analysis, treatment recommendations, academic simulated exams, and patient education. Research has shown that ChatGPT provides exceptional diagnostic and treatment advice in most medical disciplines. Moreover, it has successfully passed professional exams in multiple regions and disciplines, showcasing its outstanding ability to generate patient education materials instantly. With the introduction of GPT-4, the model we use has achieved a new breakthrough in the scale of training data, and the emergence of GPT-4 Vision (GPT-4V) marks the extension of language models into the realm of images, no longer limited to text analysis and generation. This progress not only expands the application scope of artificial intelligence (AI) but also opens up new avenues for multimodal interaction and analysis. We have recently taken note of Zhu *et al*.’s comprehensive and multifaceted exploration of GPT-4V’s capabilities. The research materials were sourced from USMLE, the ChestX-ray8 database, and radiological resources from the *Radiology* journal, employing different input modes such as imaging data input, medical history input, and simultaneous input of both^1^. The authors not only investigated the processing stability of GPT-4V under multi-threaded input modes but also conducted a thorough evaluation of its diagnostic and treatment capabilities. The evaluation results indicate that GPT-4V possesses the ability to integrate clinical images with patient history for comprehensive analysis and suggest that with sufficient specialized training and fine-tuning, the model’s accuracy could be further improved. However, while technological advancements drive changes in the diagnostic and treatment patterns of clinicians, providing efficient and personalized medical services to patients is undoubtedly a mark of progress. The discussion section of the article lacks a dialectical discussion on the results of GPT-4V’s capability development and validation. As we explore the possibilities of applying AI software in clinical settings, beyond praising GPT-4V’s outstanding performance and potential clinical application prospects, it is crucial to delve into the potential risks and corresponding measures in its practical application^2,3^.

Firstly, we must clearly recognize that GPT-4V is a closed-source AI model. This means that the source code of GPT-4V is not publicly available, and its use, modification, and distribution are subject to strict legal and licensing constraints. The closed-source nature presents a series of challenges, particularly when users attempt to understand the model’s internal logic and data processing mechanisms. Due to the lack of access to the underlying code, users cannot effectively explore and comprehend how its algorithms specifically operate. This opacity poses difficulties in addressing potential issues that may arise with GPT-4V. For instance, when the model generates hallucinations, produces errors, or exhibits instability, users often struggle to conduct an in-depth analysis of the causes behind these problems because they cannot directly examine the specific manner in which data is processed or the decision-making paths of the algorithms. This limits users’ ability to make rapid and targeted adjustments or optimizations when issues are identified.

Furthermore, the characteristics of closed-source models also exacerbate the trust crisis in AI systems, making it challenging for GPTs to pass review and approval by relevant ethics committees for clinical applications. The standardized usage policies, accountability systems for AI software, and traceability functions for historical usage records often lag behind the development and iteration of the software^4^. The article mentions that when using GPT-4V, guiding questions are required to prevent the system from refusing to provide diagnostic and treatment recommendations^1^. Additionally, in applications involving disease diagnosis and treatment processes, if the model misses or misdiagnoses a condition, the accompanying disclaimer implies that users must bear the responsibility themselves. If GPT-4V is used as an independent tool for processing large-scale disease screening data without final review by professionals, the issue of responsibility for missed diagnoses may pose significant legal and ethical risks. Therefore, even with the adoption of advanced AI systems like GPT-4V, it is essential to ensure the involvement and supervision of professional medical personnel in actual applications^5^. This allows for timely correction of potential errors and final confirmation of diagnostic results. Ensuring the rationality, safety, and ethics of applying AI technology in the clinical application and scientific research domain is a pressing issue that requires attention and resolution in the current development of AI technology.

Lastly, the application of LLMs like the GPT series in the medical field provides powerful support by automating the processing of large amounts of data and routine queries, helping experienced doctors break free from daily tedious and repetitive tasks^6^. This allows doctors to allocate more time and energy to complex medical tasks that require deep professional knowledge and human judgment, such as developing personalized treatment plans or handling intricate clinical situations. However, for young clinicians just entering the workforce, relying on LLMs may also pose potential risks. While these tools are highly efficient in providing information and handling routine tasks, over-reliance may undermine young doctors’ opportunities to accumulate clinical experience through practice. The cultivation of clinical skills not only requires theoretical knowledge but also relies on face-to-face patient interactions and hands-on experience in executing medical procedures. Currently, there is a lack of long-term clinical trials to assess the role of LLMs in medical education, particularly longitudinal studies on their impact on the professional development of young doctors. Future research needs to explore how to balance the use of LLMs with the development of doctors’ individual skills, ensuring that these technological tools enhance the quality of medical services without hindering the comprehensive growth of doctors’ professional skills.

In conclusion, Zhu *et al*.^1^ conducted an in-depth exploration and analysis of GPT-4V’s ability to perform image reading by integrating clinical information. Their research findings not only provide important insights for clinicians in actual application scenarios of GPT-4V but also offer strong support and evidence for developers in refining GPT-4V’s functionalities. We are excited about the development of this potential new type of clinical assistance tool, which signifies a major advancement in medical practice. However, as this advanced technology is introduced, we must also more carefully scrutinize and address the accompanying ethical issues, including data privacy protection, patient information security, and the transparency of AI decision-making processes. Moreover, ensuring that these technologies do not exacerbate healthcare disparities or the risk of misdiagnosis is also a critical challenge we must confront. Therefore, while developing these technologies, it is imperative to establish a strict regulatory framework and ethical guidelines to ensure that all clinical applications are conducted within the bounds of ethics and the law.

## Ethical approval

None.

## Consent

No consent is required for this commentary.

## Sources of funding

None.

## Author contribution

The authors declare that they have made equal and significant contributions to this work. The authors have approved the final version of the manuscript and agree to be accountable for all aspects of the work.

## Conflicts of interest disclosure

There are no conflicts of interest.

## Research registration unique identifying number (UIN)

Not applicable.

## Guarantor

Zhiling Zhang; e-mail: huansongwei123@163.com

## Data availability statement

The data underlying this article will be shared by the corresponding author on reasonable request.

## Provenance and peer review

We are not invited.

## Assistance with the study

None.

## Presentation

None.
